# Judicial judgment and media sensation of violence against medical staff in China: A fuzzy set qualitative comparative analysis (fsQCA)

**DOI:** 10.1371/journal.pone.0259014

**Published:** 2021-10-22

**Authors:** Jian Xu, Yongrong Cao, Yangyang Wang, Qingquan Qiao

**Affiliations:** 1 School of Media and Communication and China Institute for Urban Governance, Shanghai Jiao Tong University, Shanghai, China; 2 School of Media and Communication, Shanghai Jiao Tong University, Shanghai, China; 3 China Institute for Urban Governance, Shanghai Jiao Tong University, Shanghai, China; University of Defence in Belgrade, SERBIA

## Abstract

**Introduction:**

Violence against medical staff has been prevalent in China over the past two decades. Although Chinese authorities have released many laws and regulations to protect medical staff from violence since 2011, the legal approach alone is unlikely to resolve this complex issue. In particular, several cases of violence against medical staff in China have caused great media sensation.

**Method:**

This paper proposes an integrated model that combines the environmental stimuli theory, broken windows theory, and rational choice theory. It adopts the fuzzy set qualitative comparative analysis (fsQCA) to untangle the causal relationship between violence against medical staff, media sensation, and judicial judgment. We examined reports of medical violence on media and news websites from January 1, 2010, to January 31, 2020, and selected 50 cases with detailed information for this study.

**Results:**

The results show that each condition is not sufficient for the absence of *judicial judgment*, but when combined, they are conducive to the outcome. The conditions of *hospital level*, *medical cost*, and *media sensation* play important roles. The providers, patients, and environmental factors are indicators of inadequate or lack of *judicial judgment*, which corresponds to previous expectations.

**Conclusions:**

The integrated model greatly enriches the extant theories and literature, and also yields implications for preventing violence against medical staff in China. We suggest that sustainable and innovative healthcare reform should be initiated. For example, public hospitals should remain the cornerstone of national public health security. Medical staff in public hospitals must be regarded as “civil servants”. Therefore, the current legal system should be improved. The media should objectively report events concerning medical staff and improve public healthcare knowledge.

## Introduction

In the past two years, there have been several sensational media reports on violence against medical staff in China. On December 24, 2019, Yang Wen, an emergency physician and expert of hypertension disease at the Civil Aviation General Hospital in Beijing, was stabbed and killed by a patient’s son. The atrocity was strongly condemned by the Chinese Medical Doctor Association (CMDA) and triggered an outpouring of anger among the public. Twenty-three days later, the suspect was sentenced to death by the Beijing No. 3 Intermediate People’s Court. Yang’s case attracted huge attention online, with 2.5 billion views of related hashtags on social media [1]. The Baidu Index (http://index.baidu.com/) contains 101,000 relevant news items [2]. Since January 24, 2020, more than 42,000 medical staff from China’s 31 provincial-level regions have gathered in Wuhan to fight the Corona Virus pandemic (COVID-19). However, cases of violence against medical staff repeatedly took place and caused media sensation. For example, on January 29, 2020, two doctors were beaten and had their hazmat suits and face masks ripped open in the quarantine units by a patient’s relative after the patient died from COVID-19. The violence led to the two doctors becoming infected with the coronavirus, and relevant news items on the Baidu index reached 1.13 million. In a similar case a few days later, several patients took off some doctors’ masks, coughed heavily, and spit deliberately toward the doctors. One of the patients tore off the doctors’ protective clothing and shouted, “why do you wear protective clothing? Let’s die together”.

To prevent violence against medical staff from further increasing, authorities from the central government have released more than ten laws and regulations since 2011 [3]. This shows China’s legislative determination to protect medical staff from violence. For example, on December 28, 2019, just a few days after the Yang Wen incident, the Chinese government introduced *the Basic Health Care*, *Medicine and Health Promotion Law* and announced that it would take effect on June 1, 2020. China’s National Health Committee (NHC), Supreme People’s Court (SPC), Supreme People’s Procuratorate (SPP), and the Ministry of Public Security (MPS) jointly released an emergency regulation on February 8, 2020, to protect medical staff fighting COVID-19 from possible violence and assault [4]. Although these laws and regulations reveal enough official benevolence toward medical staff, the legal approach alone “is unlikely to adequately address this complex problem” [5]. This is especially true when the Chinese judicial system is often criticized for its poor performance in dealing with medical disputes. Previous solutions to many cases of medical malpractice, disputes, and violence have been criticized for lacking procedural justice or third-party supervision and petition [6]. In reality, this malpractice facilitates the spread of medical disturbance and violence by people who “always initiate malignant incidents, public instability, and even the criminal cases” and “purposefully evade the normalized way to resolve disputes with illegal approaches” [7].

Previous research suggests that multiple factors contribute to violence against medical staff. They include providers (e.g., defensive medicine, insufficient communication), patients (e.g., distrust of doctors and hospitals), environment (e.g., biased media), and government (e.g., unfair judicial system) [8, 9]. In this paper, we aim to untangle the causal relations between violence against medical staff, media sensation, and judicial judgment. We believe that providers, patients, and environment factors exert influence on judicial judgment (government factors), further escalating violence against medical staff. Our research questions are as follows: whether provider factors (e.g., hospital and cost), patient factors (e.g., disease, perpetrator, weapon, and injury), and environmental factors (e.g., media sensation) are indicators of the inadequate or lack of judicial judgment. We adopt a qualitative comparative analysis (QCA) to answer these questions and identify the possible conditions causing a lack of judicial procedures.

## Literature review

Even though medical staff (including doctors, nurses, and other personnel) are often compared to “angels” [10], violence against them (“Yinao (医闹)” in Chinese) has been a global concern [11, 12]. Violence against medical staff could be either physical (e.g., assault/attack and homicide) or psychological (e.g., verbal abuse, bullying/mobbing, sexual harassment, and threats) [13]. It has become one of the most common problems in both developed and developing countries [14]. Cebrino and Portero de la Cruz (2020) conducted a worldwide bibliometric analysis of the published literature on workplace violence in healthcare personnel from 1992 to 2019. Their analysis shows that publications have grown steadily each year, reaching a total of 1791 records from 85 different countries and regions. The United States produces the most publications by far, totaling 549. Australia and the United Kingdom produced 183 and 110, respectively. Four countries produce between 50 and 100 publications (ranked decreasingly as Canada, China, Italy, and Turkey), and 78 countries produce 50 or fewer publications [15]. In general, compared to doctors, nurses are more likely to be exposed to violence and aggression from patients and their families/friends worldwide, and verbal violence is the most common form of violence, followed by threats, physical assault, and sexual harassment [16].

Ramacciati et al. (2018) conducted a narrative review of theories and frameworks on violence against emergency nurses [17]. Analyzing 459 articles worldwide, they found that 24 theories and frameworks pertain to this issue. First, they selected several theories to explain violence toward emergency nurses.1) The broken windows theory contends that if emotional abuse is tolerated in work environments (e.g., hospital), outsiders to the organization (e.g., patients) are more likely to become increasingly violent and aggressive [18]. 2) The rational choice theory assumes that offenders are reasoning actors who weigh perceived risks or potential pain (e.g., punishment through a system of laws) and rewards or benefits (e.g., enjoyment) associated with committing a crime [19]. 3) The environmental stimuli theory suggests that unpleasant environmental stimuli intensify violence or aggression [20]. Second, multiple factors are considered to have contributed to violence toward emergency nurses. 1) Factors exclusively focusing on the assailant. For instance, biological theories focus on the biological, psychological, neurological, and biochemical conditions of the assailant [21]. The STAMP violence assessment framework identifies five key elements of an assailant. STAMPEDAR, as an extension of STAMP, identifies an additional four factors [22]. 2) Multiple factors were identified. For example, Poyner and Warne’s model assumes that situational and interpersonal factors are crucial, and identifies five characters (the assailant, the employee, the situation, the interaction between the assailant and employee, and the outcome) [23]. The ecological occupational health model identifies four factors: individual workers, workplace, external environment, and assault’s situation [24]. The routine activity theory regards motivated offenders, target suitability, and guarding as three major factors [25]. The social-ecological model identifies four factors: individual, relationship, community, and societal [26]. Honneth’s struggle for recognition theory identifies three main themes: unmet needs, involuntary assessment, and unsolicited touch [27].

Additionally, Angland et al. (2014) revealed the following two factors: environmental and communicational [28]. Furthermore, Ramacciati et al. (2014) categorized four causal factors considering the nurses’ viewpoint: external, internal, environmental/contextual, and organizational [29]. Martinez (2016), and Aljohani et al. (2021) identified three risk factors (patients, environment, and staff) [30, 31]. Kleissl-Muir et al. (2018) reviewed 1901 articles and identified four factors (perpetrator characteristics, substance abuse, patient-related factors, environment, and institutional factors) [32]. In summary, the abovementioned factors can be categorized into four main factors: patients (e.g., clients, perpetrator/assailant/offenders), providers (e.g., nurses/doctor/employee/target and hospital/institutions/guarding), environment (e.g., external environment and societal), and government (e.g., punishment through a system of laws).

In mainland China, the scale, frequency, and viciousness of attacks on medical staff have been particularly severe in the past two decades. The annual number of medical disputes has dramatically increased from 6,324 in 2003 to 115,000 in 2014 [8]. Medical staff are frequently abused and injured. Many have even been killed by patients or their relatives in or outside hospitals and clinics [33]. Based on the broken window theory, Zhou et al. (2017) identified three principal factors influencing workplace violence in hospitals in China (hospital-related, patient-related, social, and governmental factors) [34]. Reviewing 169 publications issued from 1998 to 2014, Geng (2015) identified four factors (providers, patients, environment, and government) that contribute to medical disputes [8]. Patient factors include high expectations, health literacy, and distrust of doctors and hospitals. Provider factors include medical malpractice, poor quality of care, negative attitude, insufficient communication with patients, and overuse of expensive services and drugs. Environmental factors include limited coverage, low competition, limited choices, and biased or negative media reports. Government factors include poor investment, ineffective supervision, and a lack of a medical damage risk-sharing mechanism [8, 9].

In the era of social media, mass media (including internet, television, print media, and radio) not only plays an essential role in communicating information about health and health services [35, 36], but also has a “detrimental impact” on the online and offline mobilization of public opinions [37–40]. Yang et al. (2021) investigated netizens’ responses to media reports on violence against doctors to examine online public opinions toward perpetrators and doctors to demonstrate whether such trends are influenced by 15 national policies in China from 2011 to 2020. They found that the proportion of “blame big system (blame for the whole society or social atmosphere)” and “blame medical system (blame for health policy or the hospital system)” are more stable. “Blame doctors” started at 33.3% in 2011, peaked at 39.2% in 2013, but decreased to 12.97% in 2014 and 4.86% in 2020. “Blame patients” started at 26.7% in 2011, reached 10.3% in 2013, increased to 24.58% in 2014, and decreased to 12.71% in 2020. “Support doctor” decreased from 2011 (25.5%) to 2012 (16.9%), but rose significantly from 2015 (12.7%) to 2016 (33.9%). “Support patient” showed a significant downward trend from 2011(23.5%) to 2020 (2.52%). The changing online attitude is “temporally” associated with government interventions. The declarations of ten national policies were followed by increases in the proportion of “support doctors”. A decline in the proportion of “blamed doctors” followed three promulgated policies [3].

**Fig 1:**
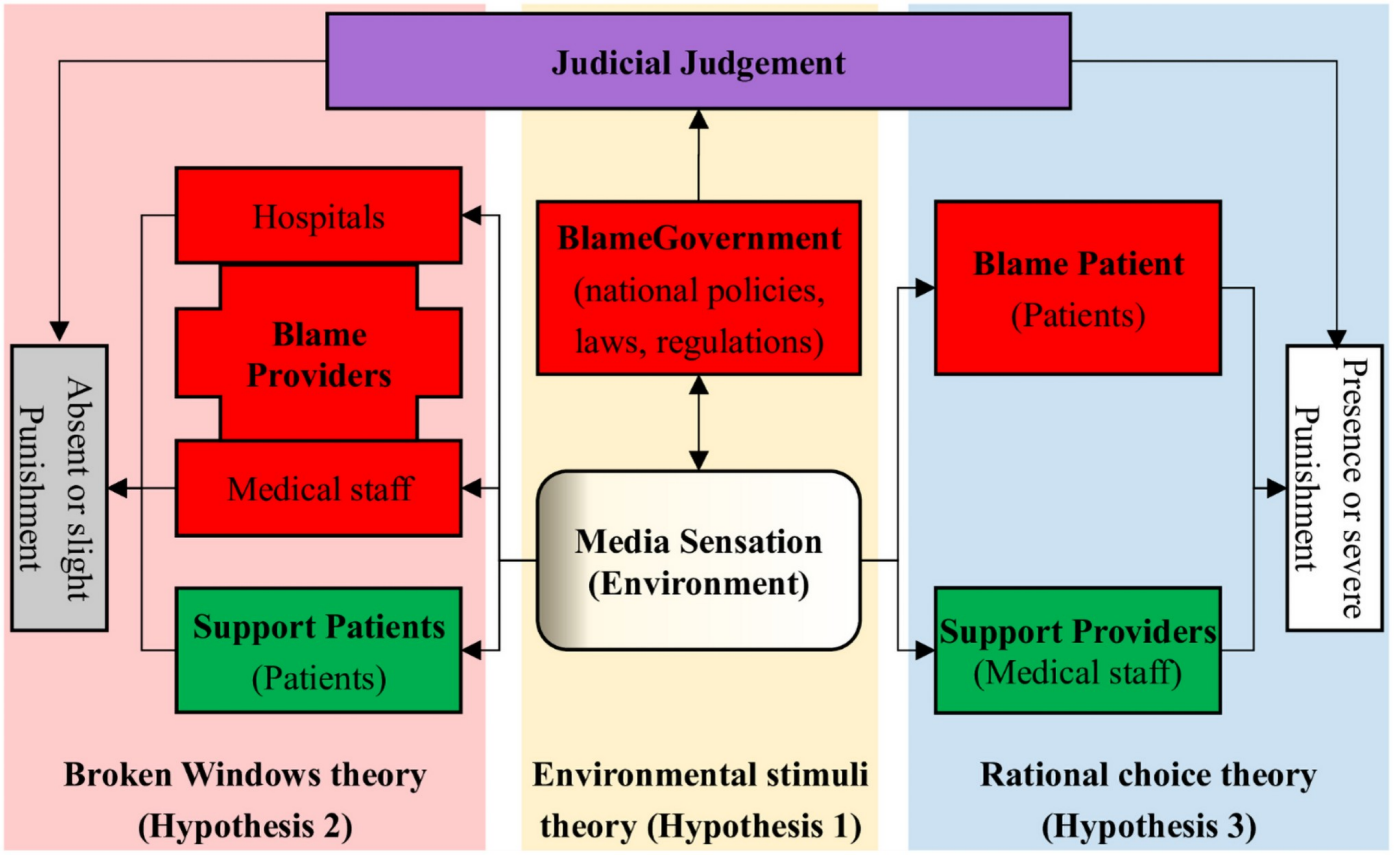
Integrated model of violence against medical staff. “Support” means support from the public opinion; “Blame” means blame from the public opinion.

Under such circumstances, resolving and preventing violence against medical staff has become much more complex and complicated. Therefore, we propose an integrated model that combines the environmental stimuli theory, broken windows theory, and rational choice theory, and includes four main factors (providers, patients, environment, and government) (see Fig 1). This research aims to explore the cause-and-effect relationship between violence against medical staff, media sensation, and judicial judgment in China. Three hypotheses (H1, H2, and H3) were developed.

Hypothesis 1 (H1): Media are environmental stimuli according to environmental stimuli theory. Media tend to sensationalize violence against medical staff [40], yet negative and biased media reports may exacerbate the tension between patients and doctors, and stimulate copycat incidents [41–44]. Tan et al. (2021) retrieved 788 eligible and domestic media reports on violence against medical providers in China from January 2007 to December 2017, and found that the majority of reports do not comply with professional journalism recommendations [41]. In generating sensation, media tend to report on medical negligence and malpractice, health system failures, and bribe-taking cases, which create public distrust and anger against medical staff [45]. By the end of 2020, the number of Chinese netizens reached one billion. Social media allow netizens to immediately share their reactions with reported stories [46]. Meanwhile, increased application of social media in medicine has even led to the development of new forms of aggression, that is, online violence against healthcare providers, such as hate speech, trolling, cyberbullying, and cyberstalking [47–50].

Hypothesis 2 (H2): The broken windows theory suggests a tendency for medical staff to tolerate violent behaviors. Cases of violence take place not only with the “tolerance” of the general public and medical providers (both hospitals and medical staff), but also with the impunity of legal authorities [51]. Negative publicity can increase online blame for providers and can cause reputational damage to them [52]. Medical staff are not always willing to report violence [30], because they either perceive violence as an inevitable part of their daily job [29, 53, 54], regard “reporting violent incidents as implying an inability to provide patient care and perform job duties competently” [55], believe it is useless to report violence “because no institutional intervention will be implemented” [56, 57], or feel that they may be unfairly criticized or punished by their superiors in the hospital [58, 59]. For the sake of reputation, hospital administrators tend to settle disputes through negotiations with patients or by financial compensation [58]. On the other hand, existing channels for reporting malpractice or resolving medical disputes are perceived as time-consuming, burdensome, complicated, unpractical, unaffordable, ineffective, and unfair [56, 58, 60–63]. Although the use of reporting systems [63–65], easy registration and time-saving systems [56], has been on the rise, under-reporting remains high [54, 56, 63]. On June 24, 2015, the SPP stated that “Procuratorate at all levels will try to promote reconciliation when the medical mobs are patients or their relatives; on the contrary, they will attack professional mobs” [66]. Analyzing 5614 cases from 2013–2015 in Guangdong Province, China, Wang et al. (2020) found that more than 90% of medical disputes were solved through mediation [52]. The different attitudes toward medical mobs and patients’ families, as shown by SPP’s statement, are mistakenly attributed to the government’s tolerance and forgiveness of violent behavior from patients and their families [66].

Hypothesis 3 (H3): Rational choice theory suggests that tougher law enforcement is needed to reduce violence against medical staff. Chinese authorities took series of measures to alleviate the intense patient-doctor relationship, and violators have to rationally calculate costs and benefits [19]. On the one hand, the Chinese central government promulgated 19 policies from 2011 to 2020 [3]. These measures include: severe punishment of any illegal activities harming the safety of doctors or patients [67], resolutely combat crimes involving hospitals to maintain order, and punish mob behavior [68]. On the other hand, without government supervision, media have led to misinformed online public opinion [34]. Thus, the Chinese government has made great efforts to regulate online content to maintain social stability both online and offline [67]. For instance, the newly amended *Criminal Law* in 2020 makes it illegal to create or spread rumors on the Internet in China [69]. Unfortunately, the government’s administrative interventions merely temporarily shaped online public opinions [3].

## Data collection and research design

**Fig 2:**
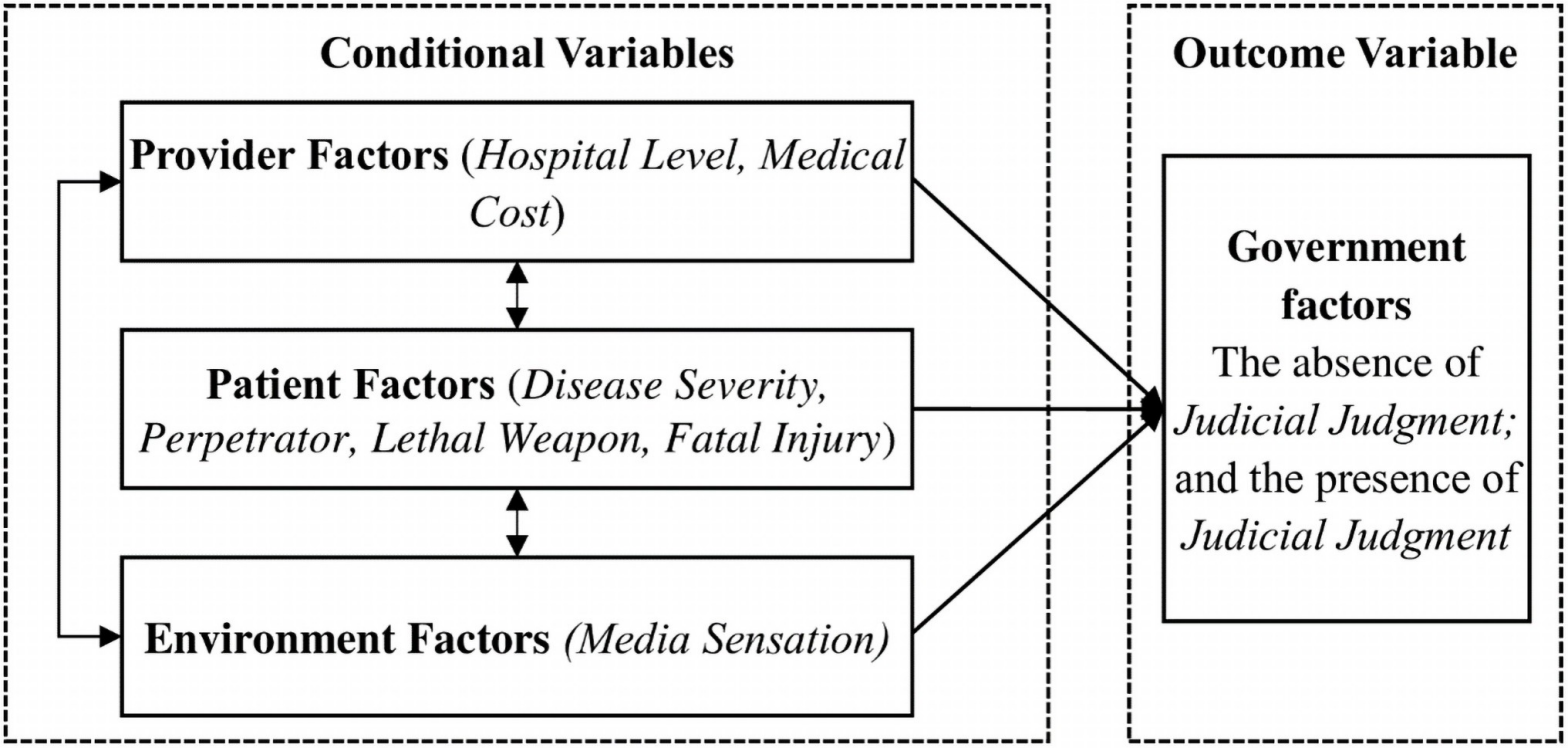
Research proposition.

Violence against medical staff in China has steadily increased over the past decade and has become a serious, ubiquitous, and persistent social problem [70]. The data included reports of medical violence on media and news websites from January 1, 2010, to January 31, 2020. We selected these cases through Baidu and Google search engines with Chinese keywords such as “violence against medical staff (暴力伤医),” “kill the medical staff (杀医),” and “hack medical staff in hospital (医院砍人)”. As a result, we obtained 50 cases with detailed information. We understand that these cases are just the tip of the iceberg among numerous cases of hospital violence, which are largely underrepresented by the media [71]. Fig 2 presents our research proposition. We aim to identify the antecedent condition of “the absence of judicial judgment”. We further carried out a qualitative comparative analysis (QCA) of the data, which was first introduced by Ragin in 1984, to test the developed propositions [72].

The outcome is represented by *a judicial judgment*. Although hospitals have established a series of preventative measures and the government has initiated stricter punishment of “Yinao,” legal channels to solve medical disputes are still lacking [73, 74]. In China, criminal litigation and civil litigation involve violence against medical staff. Cai et al. (2019) analyze 459 criminal cases involving violence against medical staff in China between 2013 and 2016 from China Judgment Online (https://wenshu.court.gov.cn/), which is operated and maintained by the SPC. They find that 77.3% involve physical abuse, followed by property damage (26.4%), disruption of public order (25.7%), verbal abuse (17.4%), and intentional homicide (3.1%); the violence injuries included death (1.3%), severe injuries (5.2%), medium injury (42.5%), minor or unhurt (32%), and missing value (19%) [70]. Li et al. (2019) collected all 140 criminal ligation records released by the SPC in China from 2010 to 2016, and found that 62.5% of patients died; physical violence involves the use of sharp instruments (9.3%), blunt instruments (12.1%), beating (37.1%), biting (7.9%), pushing (21.4%), etc.; psychological violence involves verbal abuse (24.3%), threatening (16.4%), etc.; only 8.6% chose to conduct medical malpractice technical authentication; Two perpetrators were sentenced to death for murder, and the majority (110 cases) were sentenced to fixed-term imprisonment [75]. Li et al. (2014) analyzed 1086 medical malpractice litigation data in China between 1998 and 2011 from the Peking University Center for Legal Information (http://www.pkulaw.cn/). They discovered that 77% of the cases fell into the civil judgment category, 14% into civil ruling, and 8% into civil mediation [76]. Therefore, *judicial judgment* represents judicial decisions or judgments issued by courts through civil or criminal litigation. We coded *judicial judgment* as a four-scale Likert scale: 4) death penalty (including death sentence with reprieve); 3) imprisonment (including life imprisonment); 2) detention (including criminal and administrative detention); and 1) no punishment at all (see Table 1), according to the *Criminal Law (2020 Amendment)*, *Civil Procedure Law (2017 Amendment)*, *and Criminal Procedure Law (2018 Amendment)*. All these outcomes and conditions are 4-scale Likert scale variables. Detailed information is provided in Table 1.

**Table 1:** Definitions, scale point, and code of condition and outcome.

Condition/Outcome	Variable	Scale Point	Code
Outcome	*Judicial Judgment*	4) Death Penalty; 3) Imprisonment; 2) Detention; 1) None	*Judgment*
Condition	*Hospital Level*	4) Tertiary; 3) Secondary; 2) Primary; 1) The others.	*Hospital*
Condition	*Medical Cost*	4) Above 50 thousand; 3) 10–50 thousand; 2) 5–10 thousand; 1). Less than 5 thousand	*Cost*
	*(Unit*: *RMB)*		
Condition	*Disease Severity*	4) Fatal; 3) Severe; 2) Light; 1) Slight.	*Disease*
Condition	*Perpetrator*	4) Patient; 3) Patient’s relatives; 2) Patient’s friends; 1) No relation with the doctor.	*Perpetrator*
Condition	*Lethal Weapon*	4) Lethal and prepared; 3) Lethal, non-prepared; 2) Non-Lethal, non-prepared; 1) None	*Weapon*
Condition	*Fatal Injury*	4) Death; 3) Severe injury; 2) Medium injury; 1) Minor injury or unhurt [77]	*Injury*
Condition	*Media Sensation* (measured by Baidu Index)	4) Above 100 thousand; 3) 10–100 thousand; 2) 3–10 thousand; 1) Less than 3 thousand	*Media*

Provider factors are related to hospitals and are represented by *hospital level* and *medical costs*. On the one hand, medical resources remain insufficient in China, and there is a structural imbalance between the three tiers of healthcare facilities. Data from the NHC show that China had 33,972 hospitals by the end of November 2019. Among them, public hospitals account for 35%, and private hospitals account for 65%. Tertiary, secondary, primary, and other types of hospitals account for 7.9%, 27.9%, 32.4%, and 31.8%, respectively [78]. However, patients flood into the top tier of hospitals. Within the first 11 months of 2019, Chinese hospitals received a total number of 3.4 billion patients. However, public hospitals received 2.89 billion (84.9%). Tertiary and secondary hospitals received 1.77 billion (51.8%) and 1.23 billion (36.0%) respectively [79]. This imbalance results in a long waiting time and insufficient communication between the medical staff and patients. Tertiary and secondary hospitals usually receive more patients with more severe diseases. Previous research has demonstrated that violence against medical staff is more prevalent in tertiary and secondary hospitals [70]. On the other hand, the commercialization of China’s healthcare system has witnessed a rapid increase in medical costs. Before 1985, medical disputes and violence against medical staff were rare, because nearly all hospitals were public and received full government funding. With economic reforms in the 1980s, the Chinese government spent less money on the public health industry. Public hospitals struggled to make ends meet and had to make a living and profit by transferring costs to patients. As a result, medical expenses became much more costly for the general public. In particular, patients suffering from severe diseases may spend all their savings on medical care. Conflicts usually occur when costly medical expenses and poor treatment fail to meet patients’ expectations. Thus, we expect *hospital level* and *medical costs* to be antecedent conditions of the absence of *judicial judgment*.

Patient factors included *perpetrators*, *fatal disease*, *lethal weapons*, and *fatal injury*. *The perpetrator* measures the relationship between the perpetrator, patient, and doctor. A perpetrator can be a patient or a patient’s relatives and friends. In some cases, the perpetrator may have no relationship with the doctor. There is a positive correlation between the severity of a patient’s illness and the possibility of physical violence against medical staff [80]. Some patients and their family members tend to overestimate the effects of medical interventions, believing that spending on medical treatment must translate into full recovery, even in the case of untreatable disorders. Once medical treatment does not prevent the death of patients, their families tend to blame doctors and demand a huge amount of financial compensation from the hospital. In other words, patients’ unrealistically high expectations are a major factor leading to violence [81]. The presence of weapons facilitates the spread and severity of violence. In many cases, patients and their relatives and friends are armed with weapons, such as knives and axes. These weapons are more likely to cause death or fatal injuries to medical staff. Unfortunately, it is difficult to predict who is carrying lethal weapons in particular situations [82]. Therefore, we expect the four variables in patient factors to be antecedent conditions of the absence of *judicial judgment*.

Environmental factors mainly concern *the media sensation*. Chinese media plays an important role in provoking tension between medical staff and patients and influencing public opinion about medical staff [83]. To attract public attention, some Chinese media reports exaggerate medical disputes and deliberately portray a negative image of medical staff (e.g., greedy, Hong bao, and poor qualifications). Biased and misrepresented media reports can result in a patient’s lack of trust in medical staff. Both hospitals and the judicial system are subsequently confronted with poor public opinion [73, 84]. For example, in 2010, one “fake news” reported that a new mother’s anus was intentionally stitched by the nurse because her family had not bribed the nurse in advance. Sensational reports such as this tend to distort facts, usually presenting the patient’s side of the story without giving medical staff the corresponding right of expression. We measured medical sensation using the Baidu Index. The greater the media sensation, the more audience and media coverage an incident attracts. Thus, we expect that *media sensation* is an antecedent of the absence of *judicial judgment*.

To validate the above-mentioned propositions, we employ the fsQCA analysis with the latest version of the fsQCA 3.1b software. We first perform a calibration of the conditions and outcome, which refers to “the assigning of fuzzy set values to conditions of individual cases” [85]. As for the conditions and outcome are four-scale Likert scale variables, the cases receive the following scores: 0, 1; 0.33, 2; 0.67, 3; 1, 4.

## Findings

### Descriptive statistics

**Fig 3:**
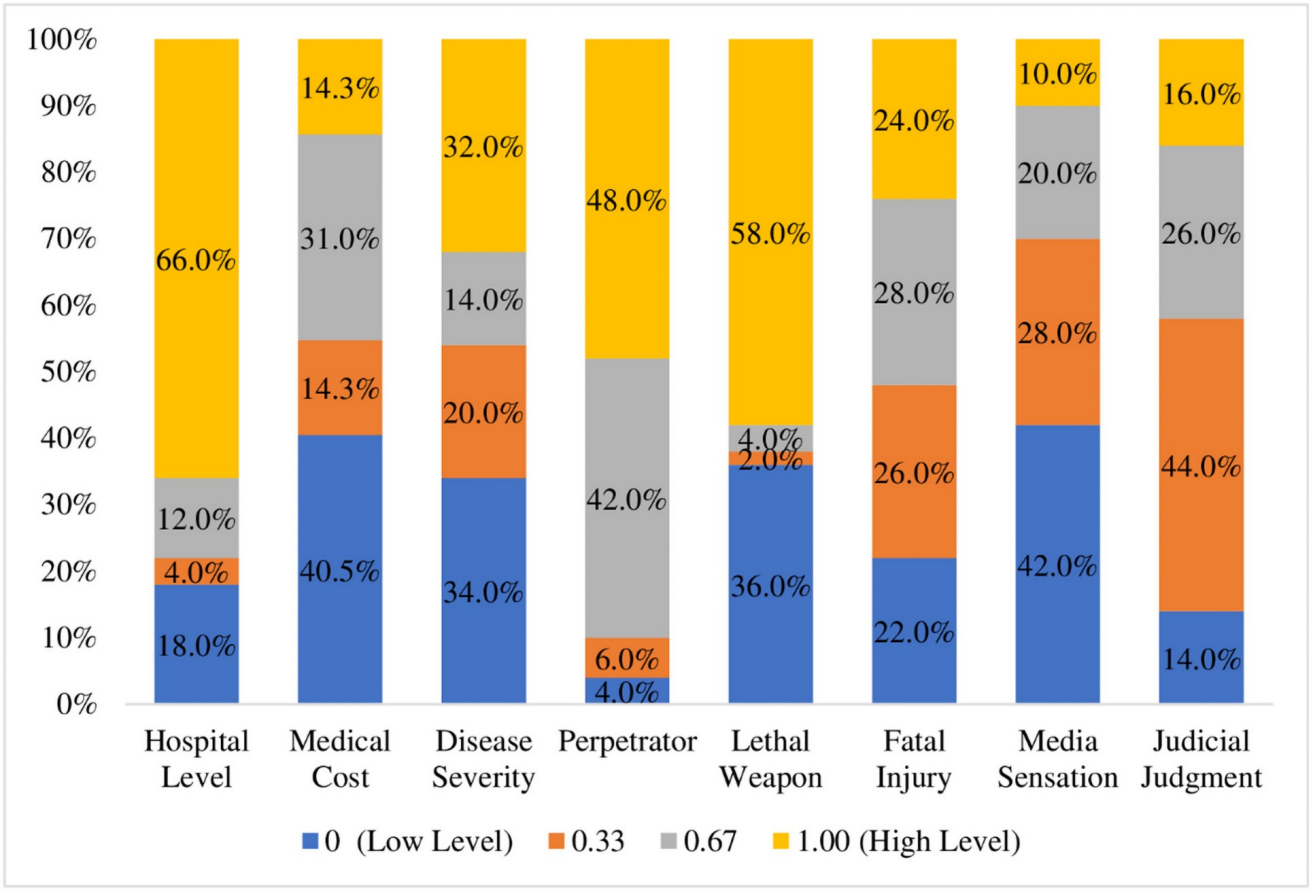
Frequency statistics for the conditions and outcome.

Fig 3 shows the frequency statistics of the conditions and outcomes. Among the 50 cases examined, 66.0% of the violence cases took place in tertiary hospitals; 40% of cases had the lowest medical cost; 32% patients’ disease was severe; 48% perpetrators were patients themselves, and 42% were patients’ relatives; 58.0% perpetrators were armed with lethal weapons; 24% suffered from fatal injury; 42% were less sensational; and 44% perpetrators were sentenced to detention. In addition, 94% of victims were doctors, and 6% were nurses [69]. The descriptive statistics for the conditions and outcomes are reported in Table 2, based on the calibrated scores.

**Table 2:** Descriptive statistics for the conditions and outcome.

Variable	Mean	Std. Dev.	Minimum	Maximum	N
*Hospital Level (Hospital)*	0.7536	0.3877	0	1	50
*Medical Cost (Cost)*	0.4208	0.3709	0	1	50
*Disease Severity (Disease)*	0.4798	0.4176	0	1	50
*Perpetrator (Perpetrator)*	0.7812	0.2546	0	1	50
*Lethal Weapon (Weapon)*	0.6134	0.4732	0	1	50
*Fatal Injury (Injury)*	0.5134	0.3611	0	1	50
*Media Sensation (Media)*	0.3264	0.3372	0	1	50
*Judicial Judgment (Judgment)*	0.4794	0.3079	0	1	50

### Necessity and sufficiency analysis

According to Ragin, “it is often useful to check for necessary conditions before conducting the fuzzy truth table procedure” [86]. Necessary conditions are “those that must be present but alone are not sufficient to produce the outcome of interest,” while sufficient conditions “are sufficient but not necessary (because of multiple causal pathways) to produce the outcome of interest” [87]. In fsQCA analyses, consistency scores above 0.9 indicate the presence of an indispensable condition for producing the outcome of interest [88]. Ragin proposes 0.80 for causal factors that are “almost always” necessary or sufficient, and 0.65 for causal conditions that are “usually” necessary or sufficient [89]. By analyzing Table 3, the necessity analysis of the absence of *judicial judgment* shows that no condition meets this threshold of 0.9 (see Table 3), and consistency scores range between 0.295 and 0.885. However, the necessity analysis of the presence of *judicial judgment* shows that *the perpetrator* meets this threshold; without this condition, the result cannot be produced, and the consistency scores range between 0.249 and 0.903. Superscript (^a^) indicates that these conditions meet or exceed the consistency benchmark of 0.65. The left side of Table 3 shows that a higher level of *hospital level*, *perpetrator*, and lower level of *medical cost*, *fatal injury*, and *media sensation* appear to be important enabling conditions for the absence of *judgment*, *disease severity*, and *lethal weapon* do not seem to be strong enablers for the absence of *judicial judgment*. The right side of Table 3 shows that a higher level of *hospital level*, *perpetrator*, *lethal weapon*, and *fatal injury*, and lower levels of *medical cost* and *media sensation* appear to be important enabling conditions for the presence of *judicial judgment*, and *disease severity* does not seem to be a strong enabler for the presence of *judicial judgment*.

**Table 3:** Results of necessary conditions.

		~ *Judgment*		*Judgment*	
Condition		Consistency	Coverage	Consistency	Coverage
*Hospital*	*Hospital*	**0.756** ^a^	0.522	**0.8060** ^a^	0.5127
	~ *Hospital*	0.295	0.623	0.2491	0.4846
*Cost*	*Cost*	0.551	0.682	0.5131	0.5846
	~ *Cost*	**0.664** ^a^	0.597	**0.7209** ^a^	0.5967
*Disease*	*Disease*	0.550	0.597	0.6099	0.6094
	~ *Disease*	0.640	0.641	0.5966	0.5498
*Perpetrator*	*Perpetrator*	**0.885** ^a^	0.590	**0.9028** ^a^	0.5540
	~ *Perpetrator*	0.331	0.787	0.3312	0.7258
*Weapon*	*Weapon*	0.577	0.489	**0.6946** ^a^	0.5429
	~ *Weapon*	0.461	0.621	0.3467	0.4299
*Injury*	*Injury*	0.588	0.596	**0.7630** ^a^	0.7125
	~ *Injury*	**0.716** ^a^	0.767	0.5674	0.5590
*Media*	*Media*	0.434	0.692	0.4714	0.6924
	~ *Media*	**0.807** ^a^	0.624	**0.7902** ^a^	0.5624

~ represents the absence of a condition or the logical operation “NOT”

^a^ Meets 0.65 consistency benchmark for usually necessary conditions.

Source: Processed with the fsQCA 3.1b software.

We test sufficiency using the truth table, which indicates the sufficient conditions for the result [90]. The fuzzy truth table algorithm incorporates a two-step analytic procedure: 1) the fuzzy data are used to create a truth table of the data matrix with 2^k^ rows (where k represents the number of causal conditions, and 2 represents the presence or absence of conditions), the 0s and 1s indicate “the different corners of the vector space defined by the fuzzy set causal conditions”; 2) specify the causal conditions and outcomes to minimize, raw consistency scores below 0.8 indicate substantial inconsistency [72]. In this study, sufficiency analysis was performed by testing the presence of the conditions that lead to the absence of the outcome within the truth table (see Table 4).

**Table 4:** Truth table (outcome: The absence of *Judgment*).

*Hospital*	*Cost*	*Disease*	*Perpetrator*	*Weapon*	*Injury*	*Media*	*number*	*~Judgment*	*raw consist*.	*PRI consist*.	*SYM consist*
1	0	0	1	0	0	0	5	1	0.888	0.818	0.818
1	1	1	1	1	1	1	5	0	0.767	0.502	0.502
1	1	1	1	1	1	0	4	0	0.529	0	0
0	1	1	1	0	0	0	3	1	1	1	1
1	1	1	1	0	0	0	3	1	1	1	1
1	0	0	1	1	0	0	3	1	0.843	0.333	0.333
1	0	0	1	1	1	1	3	1	0.814	0.333	0.333
1	0	0	1	1	1	0	3	0	0.665	0.169	0.169
1	0	0	1	0	1	0	2	0	0.798	0.599	0.599
0	1	1	1	1	1	0	2	0	0.571	0.254	0.254
0	1	0	1	1	0	0	1	1	1	1	1
1	1	1	1	1	0	0	1	1	1	1	1
0	0	0	0	1	1	0	1	1	1	1	1
1	0	0	0	1	1	0	1	1	1	1	1
1	0	0	1	1	0	1	1	1	1	1	1
1	1	1	1	1	0	1	1	1	1		1
0	1	1	1	1	1	1	1	1	1	1	1
1	0	0	0	0	0	0	1	1	0.889	0.752	0.752
1	1	0	1	1	1	0	1	1	0.875	0.507	0.507
1	1	0	1	0	0	0	1	1	0.858	0.670	0.670
0	0	0	1	1	1	0	1	1	0.858	0.670	0.670
1	1	1	0	1	1	0	1	1	0.829	0	0
1	0	1	1	0	0	1	1	0	0.795	0	0
1	1	0	1	1	1	1	1	0	0.795	0	0
1	0	0	1	0	0	1	1	0	0.747	0.496	0.496
0	0	0	0	0	0	0	1	0	0.496	0	0
0	0	1	1	0	0	1	1	0	0	0	0

Source: Processed with the fsQCA 3.1b software.

### Standard analysis

We conducted a standard analysis to create an intermediate solution in the absence of *judicial judgment*. Consistency and coverage are important in fsQCA analysis, ranging from 0 to 1 [91]. All the conditions were maintained for all analyses. Table 5 shows that the intermediate solution has a consistency value of 0.791, which surpasses Ragin’s recommended threshold of 0.75, and a coverage value of 0.871, which represents exploratory power and has a meaning similar to R-square values in regression analyses [88, 92]. Generally, the minimum criterion of coverage is between 0.25 and 0.65. There are 13 configurations in the group, leading to a negative level of *judicial judgment*, and all of them have strong consistency. This indicates that the solution is strongly related to the observed outcome. It is remarkable that eight of the 13 configurations (6–13) represent no unique coverage. This means they are not quite relevant to the overall findings and thus have not been included in later analyses [93, 94].

**Table 5:** Intermediate solution of the absence of *Judgment*.

Model: *~Judgment* = f(*Hospital*, *Cost*, *Disease*, *Perpetrator*, *Weapon*, *Injury*, *Media*);											
Algorithm: Quine-McCluskey; frequency cutoff: 1; consistency cutoff: 0.814											
	Configurations	*Hospital*	*Cost*	*Disease*	*Perpetrator*	*Weapon*	*Injury*	*Media*	raw coverage	unique coverage	consistency
A1	Hospital*~Injury*~Media	●					○	○	0.511	0.142	0.888
A2	Cost*~Injury		●				○		0.397	0.065	0.940
A3	~Perpetrator *Injury				○		●		0.254	0.013	0.869
A4	Cost*~Disease*~Media		●	○				○	0.229	0.013	0.900
A5	~Cost*Weapon*Media		○			●		●	0.204	0.051	0.760
A6	~Perpetrator*Weapon				○		●		0.166	0	0.812
A7	~Hospital*~Cost*Injury	○	○				●		0.140	0	0.787
A8	~Hospital*~Disease*Weapon	○		○		●			0.115	0	0.901
A9	~Hospital*~Cost*Weapon	○	○			●			0.115	0	0.751
A10	~Hospital*~Disease*Injury	○		○			●		0.115	0	0.819
A11	~Hospital*Weapon*Media	○				●		●	0.076	0	1
A12	~Hospital*Injury*Media	○					●	●	0.076	0	1
A13	~Hospital*Cost*Media	○	●					●	0.064	0	0.834
	Number or ●	1	3	0	0	4	5	4			
	Number or ○	7	3	3	2	0	2	2	Solution coverage: 0.871		
	Number of empty cells	5	7	10	11	9	6	7			
									Solution consistency: 0.791		

Notes: Black circles (●) represent the presence of a condition, void circles (○) indicate negation, * represents the intersections of condition, and ~ represents the negation of the condition.

Source: Processed with the fsQCA 3.1b software.

The absence of *judicial judgment* exists in five complex configurations (see Table 5). Recipe A1, with the highest coverage of 0.511, is represented by a high *hospital level’s* presence in conjunction with high *fatal injury’s* and *media sensation*’s absence. Recipe A2, with a coverage of 0.397, is represented by high presence of *medical costs* in conjunction with high *fatal injury’s* absence. Recipe A3, with a coverage of 0.254, is represented by the presence of high *fatal injury* in conjunction with a high *perpetrator’s* absence. Recipe A4, with a coverage of 0.229, is represented by a high *medical cost*’s presence in conjunction with the absence of high *disease severity* and *media sensation*. Recipe A5, with a coverage of 0.204, is represented by the high *lethal weapon*’s and *media sensation*’s presence in conjunction with high *medical cost’s* absence. The consistencies of the complex configurations were 0.791 (above 0.75), indicating that they were deemed sufficient configurations.

The conditions leading to the absence of *judicial judgment* might be quite different from those leading to the presence of *judicial judgment*. Hence, we conducted an analysis of the presence of the outcome. As illustrated in Table 6, the intermediate solution has an overall consistency of 0.824 and coverage of 0.723, also indicating a reliable solution.

**Table 6:** Intermediate solution of the presence of *Judgment*.

Model: *Judgment* = f(*Hospital*, *Cost*, *Disease*, *Perpetrator*, *Weapon*, *Injury*, *Media*);											
Algorithm: Quine-McCluskey; frequency cutoff: 1; consistency cutoff: 0.854											
		*Hospital*	*Cost*	*Disease*	*Perpetrator*	*Weapon*	*Injury*	*Media*	raw coverage	unique coverage	consistency
B1	Hospital*~Cost*~Disease*Perpetrator*Weapon	●	○	○	●	●			0.305	0.028	0.757
B2	Hospital*~Disease*Perpetrator*Weapon*Injury	●		○	●	●	●		0.291	0.028	0.873
B3	Hospital*Cost*Disease*Weapon*Injury*~Media	●	●	●		●		○	0.250	0.042	1.000
B4	Cost*Disease* Perpetrator*Weapon*Injury*~Media		●	●	●	●	●	○	0.250	0.042	0.946
B5	Hospital*Cost*Disease* Perpetrator*Weapon*~Injury	●	●	●	●	●	○		0.138	0.014	0.829
B6	~Cost*Disease*Perpetrator*~Weapon*~Injury*Media		○	●	●	○	○	●	0.097	0.083	1.000
B7	~Hospital*~Cost*~Disease*~Perpetrator*~Weapon*	○	○	○	○	○	○	○	0.055	0.055	1.000
	~Injury*~Media										
	Number or ●	4	3	4	5	5	2	1	solution coverage: 0.723		
	Number or ○	1	3	3	1	2	3	3			
	Number of empty cells	2	1	0	1	0	2	3	solution consistency: 0.824		

Notes: Black circles (●) represent the presence of a condition, void circles (○) indicate negation, * represents the intersections of condition, and ~ represents the negation of the condition.

Source: Processed with the fsQCA 3.1b software.

The presence of *judicial judgment* exists in seven complex configurations (see Table 6). Recipe B1 is represented by high *hospital level*’s, *perpetrator*’s, *and lethal weapon*’s presence in conjunction with high absence of *medical cost* and *disease severity*. Recipe B2 is represented by high presence of *hospital levels*, *perpetrators*, *lethal weapons*, and *fatal injury*, in conjunction with high *disease severity*’s absence. Recipe B3 is represented by high presence of *hospital levels*, *medical costs*, *disease severity*, *and lethal weapon*, in conjunction with high *media sensation*’s absence. Recipe B4 is represented by high presence of *medical cost*, *disease severity*, *perpetrator*, *lethal weapon*, *and fatal injury*, in conjunction with high *media sensation’s* absence. Recipe B5 is represented by high presence of *hospital level*, *medical cost*, *disease severity*, *perpetrator*, *and lethal weapon*, in conjunction with the absence of high *fatal injury*. Recipe B6 is represented by high *disease severity*’s, *perpetrator*’s, *media sensation’s* presence in conjunction with high absence of *medical costs*, *lethal weapons*, *and fatal* injuries. Recipe B7 is represented by high absence of *hospital level*, *medical cost*, *disease severity*, *perpetrators*, *lethal weapons*, *fatal injuries*, and *media sensation*.

Overall, apart from *the perpetrator*, other conditions in the hierarchy are neither sufficient nor necessary for *judicial judgment*, but in combination with other conditions, are conducive to the presence of *judicial judgment*. Examining the complex configurations of the absence and presence of *judicial judgment* uncovered vital differences. There is at least one combination of five conditions leading to the presence of *judicial judgment*. However, only two or three conditions may lead to an absence of *judicial judgment*. The results show that perpetrators are more likely to escape rather than punished judicially.

## Discussions and theoretical contributions

The results of the fsQCA show that many configurations contribute to the absence of the outcome and that providers, patients, and environmental factors are indicators of inadequate or lack of judicial judgment.

First, the high presence of media exposure (*media sensation*) could lead to the absence of *judicial judgment*. In Recipe A5, regardless of the *fatal injury* medical staff suffer from, when the perpetrators are armed with *lethal weapon* (high presence), in conjunction with the sensationalized media reports (high presence of *media sensation*) and low *medical cost* (high absence), perpetrators are more likely to receive inadequate or lack of *judicial judgment*. The findings support our expectation (H1) that the media is one of the key environmental stimuli leading to violence against medical staff. Media reports shape and are shaped by public attitudes and interests [35, 36]. According to frame theory [95], media information is often incomplete, slanted, and influenced by the intentions of journalists, editors, or owners of specific media outlets [40, 96]. Therefore, how media frames violence against medical staff can influence the public perceptions of medical providers and their services [36, 40]. Biased media reports on violence against medical staff seldom discuss the truth behind the violence and the potential legal liability of such behaviors, which further exacerbates the conflict between patients and medical staff [97], and also directly worsens the medical working environment [66]. Furthermore, online and offline public opinions can influence the judicial system [98].

Second, provider and patient factors are the antecedent conditions for the absence of *judicial judgment*. Regarding provider factors, high presence of *hospital level* (combined with high *fatal injury*’s and *media sensation*’s absence in recipe A1), and high presence of *medical cost* (combined with high *fatal injury* absence in recipe A2; combined with high *disease severity*’s and *media sensation*’s absence in recipe A4) are key conditions leading to the absence of *judicial judgment*. Regarding patient factors, in Recipe A3, high *fatal injury*’s presence and high *perpetrator*’s absence could lead to the absence of *judicial judgment*. The findings indicate that medical providers tend to settle disputes outside of judicial procedures, as expected with H2. If a laissez-faire attitude toward a harmful phenomenon in the environment is taken, people will follow such, which will intensify [34]. Most hospitals regrettably terminate disputes monetarily [76]. To avoid disputes, some of them have even formulated unfair rules, such as “a doctor or nurse will be punished for a complaint or dispute, no matter whether he or she is at fault” [66]. Apart from the lack of effective measures to protect them from violence, medical staff may be at risk of cyberbullying or hospital punishment, thus prioritizing quick resolution of disputes [48, 58, 59, 66]. Consequently, they often pay a substantial amount of money for little or no negligence. As patients and their families realize they can obtain more money quickly through threats and violence, litigation has become less popular [66]. Du et al. (2020) conducted patient surveys in 12 leading public hospitals in five Chinese provinces with 5556 participants and found that patients’ potential approaches to medical disputes are “complaining to the hospital or the health department” (32.5%), “seeking legal help” (26.3%), “direct negotiation with doctors” (19.6%), “exposing to the news/media” (9.7%), “negotiating with a third party” (7.4%), “complaining to friends and relatives” (3.0%), and “resorting to violence” (1.5%) [98].

Third, the presence of *judicial judgment* requires more conditions than the absence of *judicial judgment*. Both high *perpetrators’* and *lethal weapons’* presences appear in five of the seven recipes (see Table 6). The presence of both high *hospital level* and *disease severity* appears in four of the seven recipes. To a certain degree, Chinese authorities are attributed to the government’s tolerance and forgiveness of violent behaviors by patients and their families, as we expected with H3. According to rational choice theory, perpetrators weigh the potential pain and rewards of committing a crime [19]. Although patients and their families are responsible for most incidents of violence against medical staff [99], the proliferation of medical mobs is also induced. Encountering medical disputes with patients, medical providers in China (both hospitals and medical staff) have always adopted an evasive and conciliatory attitude and preferred to resolve disputes with money. This attitude encourages medical mobs. These mobs aim to create chaos, but seldom cause fatal harm to medical staff. It has been proven quite effective in resolving the medical disputes that the amount of compensation or indemnity depends on the extent of trouble caused by the medical mobs rather than the extent of the medical malpractice damage [39]. Some studies show that merely 5.4%-25.3% of medical disputes have been solved through litigation [66]. Lax enforcement and reticence to punish perpetrators will worsen the situation. Tougher laws on violence against medical staff are desired worldwide [100, 101]. Once violence behaviors against medical staff are seen as unacceptable and face severe punishment, potential perpetrators would reconsider the cost of their criminal activities [46].

### Theoretical contributions

In the era of social media, many countries encounter problems similar to those in China. In this study, we propose an integrated model and enrich the extant theories of violence against medical staff. We hope that it will be applicable to situations outside China.

First, violence against medical staff is a serious issue worldwide [17]. Public attention to such violence takes the form of official reports, media stories, and national initiatives in the United States [12], the UK [102]; Kenya [103], Ethiopia [104]; Turkey [105], Iran [106], India [107], Indonesia [108], and Latin America [109], etc. The flourishing development of social media not only provides constant and endless streams of news and events [46], but also spread news stories of these events broadly and quickly [41, 110]. Media worldwide tend to sacrifice clear and balanced news reporting to catch readers’ and audiences’ attention [41, 111]. Thus, social media plays a negative role by triggering violence against medical staff from patients and their relatives [36, 44, 73]. Meanwhile, medical staff are not always willing to report violence and tend to accept their physical and psychological suffering with silence. Medical providers tend to settle disputes quickly, fearing possible damage to their reputations [52]. Therefore, tougher laws on violence against medical staff were appealed. For instance, the Indian Medical Association (IMA) and Indian experts called for an “amendment to the law to curb violence against doctors” [101, 112]. The UK government issued a policy to condemn unjustified physical assaults on doctors [113]. UK experts called on tougher penalties and new laws to improve medical staff’s working conditions [114]. Experts in the US also called on tougher laws to punish violence against medical staff [115].

Second, the theoretical contributions of this article are: 1) the broken windows theory, environment stimuli theory, and rational choice theory. They have been identified as effective theories and frameworks to explain violent behaviors toward emergency nurses. However, in the era of social media, the environment of healthcare has become more complicated than ever before. Media have a detrimental impact on public views and mobilization of online and offline public opinion [37–40]. Therefore, we developed an integrated model that combines the environmental stimuli theory, broken windows theory, and rational choice theory. 2) The majority of literature on violence against medical staff regards patients, environment, and staff as risk factors [30, 31], yet only a few regard government factors as risk factors [8, 9]. Our research further proves that government factors are key risk factors for violence. The government should take solid measures to prevent medical staff from violence from patients and their families. 3) Our research also demonstrated that the problem of violence against medical staff could be “effectively faced only with a ‘multi-dimensional’ analysis of the operating ambiences and interventions with a ‘multi-directional,” considering the “complexity of the phenomenon” and the “strong interrelation between various factors” [29, 116]. Therefore, a comprehensive approach should intervene “concurrently on the training of nurses, organizational aspects, hospital architecture, security support, effective policies of zero tolerance and more” [29].

## Conclusion and implications

The research has not only greatly enriched the extant theories and literature, but also yields implications for preventing violence against medical staff in China. In 2020, when China’s national public health security was threatened by the COVID-19 pandemic, the whole society witnessed the absolute necessity and indispensability of medical staff in combating the epidemic. After the official announcement of the coronavirus threat as a first-level public health emergency, all public hospitals and their staff have been involved in this battle. More than ten thousand Chinese medical staff have risked their lives and volunteered to fight the coronavirus outbreak. They were overwhelmed, under-equipped, exhausted, and even lost their lives (e.g., Li Wenliang and Xia Sisi) [117]. They displayed an image radically different from the negative stereotypes maintained by the media in the past two decades. Therefore, it is time for the Chinese government to take this opportunity to initiate a sustainable and innovative healthcare reform to solve the long-lasting issue of violence against medical staff.

First, the government should reform its public health sector and maintain public hospitals as the cornerstone of its health system. Private hospitals have long been widely criticized for their insatiable pursuit of profit and absence in the current battle against the coronavirus in Wuhan. For example, the “Wei Zexi” case in 2016 kindled a “national outrage” against the Putian network of 8,000 facilities. Public hospitals’ heavy burden of fulfilling all kinds of tasks to make profit reveals their growing resemblance to private hospitals. This is partly the cause of the growing out-of-pocket costs and blatant conflicts between hospitals and patients [118]. In particular, Wuhan’s public hospitals accounted for 27.1% (in 2017), and its fight against the coronavirus epidemic revealed a serious shortage of medical resources (both qualified hospitals and medical personnel). Therefore, we suggest that: 1) different from market-oriented or profit-driven private hospitals, public hospitals should be purely non-profit organizations; 2) each region (at county, city, or province-level) should maintain adequate public hospitals to secure public healthcare security; 3) the government should provide sufficient public funding for public hospitals, as at present, China has 20% of the world’s total population, yet its health expenditure only accounts for 3% of the world’s total; and 4) raise the social and economic status (e.g., salary) of medical staff, and strengthen their professional ethics and skills of communication, etc. In particular, the phenomena of “red envelopes” and “defensive medicine” should be forbidden [119].

Second, medical staff in public hospitals should be regarded as “civil servants”. On April 26, 2019, Xiaojing Zhao, a well-known thoracic surgeon, was dragged away in handcuffs and forcibly removed from a hospital in Shanghai because of disputes with patients. Police asked him to the police station for further investigation, but he refused for “having patients and then meetings”. Later, the CMDA stated that doctor-patient conflicts should not be treated as “a typical civil dispute”. However, the *China Daily* debated that medical staff should not be “superior” to “other people” [120]. Zhao’s case illustrates that China’s medical staff have low social respect. The Wuhan epidemic demonstrates to us that medical staff shoulder the important responsibility to heal the wounded and save patients’ lives, and they are as important as firefighters and policemen. Guaranteeing them a safe working environment means they can better safeguard patients’ health and life. The Chinese government should establish regulations to guarantee the status of medical staff as “civil servants”. The authorities should be obligated to protect their work from interruption and to ensure that similar cases to Zhao’s are not repeated.

Third, any type of violence against medical staff should be forbidden and strictly punished. To a certain degree, the provider, environment, and government factors encourage violence against medical staff. Hospital leaders, police authorities, and judicial departments should adopt “zero tolerance’ attitudes toward perpetrators (patients or their relatives and friends). Hospitals tend to settle disputes through negotiations or financial compensation. Law enforcement professionals tend to underestimate medical violence as simply a “doctor-patient relationship problem” rather than criminal behavior [61, 121]. Patients hire gangs to blackmail hospitals for large amounts of financial compensation without having to worry about possible legal punishments. Violence against medical staff has even turned into a “profitable industry,” and some people take it as their career. Therefore, there has long been a rule of “allocating medical resources according to the violence”. For instance, in Yang Wen’s case, the hospital compensated the mother of the perpetrator with “special services”. “Zero tolerance of violence” needs to be more than just a slogan. Actions must be strictly taken to protect the country’s medical workers. We should 1) improve the current legal system to effectively process medical disputes, 2) rely on a stricter enforcement of relevant laws and regulations, and 3) relevant government institutions should evaluate medical disputes in a professional and legal manner.

Fourth, the media should portray medical staff in an objective manner and help eliminate health illiteracy. Within the current social context, the mass media is key to the formation of public opinions on doctor-patient relationships [122]. However, in the past two decades, the media has portrayed the image of medical staff as greedy, having a bad attitude, and lacking professional ethics [42]. Health literacy is “the ability to understand basic health information and make appropriate decisions” [123]. Due to health illiteracy, patients tend to hold unrealistic expectations of doctors [124], which may turn into animosity and desperation once expectations are not met [70]. When dissatisfied and indignant patients are confronted with exhausted and impatient doctors, the likelihood of conflict increases [125]. The legal system and public media should play a key role in improving the social environment. In the media, legal perspectives relating to medical disputes are inadequately represented, and constructive measures for protecting doctors and preventing hospital violence are rarely discussed [84]. Relevant government bodies need to use the media to disseminate core concepts about health literacy, so as to raise health literacy of the general public by improving both the access to and quality of information [126]. Medical literacy education could spread the common sense that doctors are not omnipotent and that the death of patients is irreversible in certain circumstances. Chinese medical staff fighting against COVID-19 in 2020 has demonstrated that they are in “a grand and noble career”. It is time for the Chinese media to get rid of their bias and form a positive image of medical staff. The government should supervise media to ensure the accuracy and authenticity of media coverage [127]. Any biased, unfair, or false report of medical staff in the media must be prohibited and severely punished.

## Limitations and future research

This study had several limitations. First, we collected reports on violence against medical staff available on media and news websites only from January 1, 2010, to January 31, 2020. In fact, with the new millennium, medical disputes have become a major issue in China. There have been numerous reports of medical staff being attacked by patients and their family members [62]. Although the Chinese government enacted the *Regulation on the Handling of Medical Accidents* in 2002, the annual incidents of medical disputes increased dramatically by more than 18 times from 2003 to 2014 [8]. Second, this study was set in the Chinese context, with merely 50 cases. As a result, we expect some bias in our findings. In our future research, on the one hand, we plan to collect those eligible cases with details from 2000 to present. On the other hand, we would like to collect those cases around the world through Google search engines using English keywords such as “violence against medical staff (include in doctor, nurse, etc.)”, particularly in the five continents with both developed countries (e.g., United Kingdom and United States) and developing countries (e.g., India, Indonesia, and Ethiopia). Finally, we plan to further prove the effectiveness and efficiency of the integrated model outside China.

## Supporting information

S1 Data (CSV)
